# Mechanochemical Preparation, Solid-State Characterization, and Antimicrobial Performance of Copper and Silver Nitrate Coordination Polymers with L- and DL-Arginine and Histidine

**DOI:** 10.3390/ijms24065180

**Published:** 2023-03-08

**Authors:** Cecilia Fiore, Andrii Lekhan, Simone Bordignon, Michele R. Chierotti, Roberto Gobetto, Fabrizia Grepioni, Raymond J. Turner, Dario Braga

**Affiliations:** 1 Dipartimento di Chimica “Giacomo Ciamician”, University of Bologna, Via Selmi, 2, 40126 Bologna, Italy; 2 Department of Biological Sciences, University of Calgary, 2500 University Drive NW, Calgary, AB T2N 1N4, Canada; 3 Dipartimento di Chimica and NIS Centre, University of Torino, Via P. Giuria, 7, 10125 Torino, Italy

**Keywords:** antimicrobials, coordination polymers, crystal engineering, co-crystallization, silver, copper, amino acids, mechanochemistry

## Abstract

The antimicrobial activity of the novel coordination polymers obtained by co-crystallizing the amino acids arginine or histidine, as both enantiopure L and racemic DL forms, with the salts Cu(NO_3_)_2_ and AgNO_3_ has been investigated to explore the effect of chirality in the cases of enantiopure and racemic forms. The compounds [Cu·AA·(NO_3_)_2_]_CPs_ and [Ag·AA·NO_3_]_CPs_ (AA = L-Arg, DL-Arg, L-His, DL-His) were prepared by mechanochemical, slurry, and solution methods and characterized by X-ray single-crystal and powder diffraction in the cases of the copper coordination polymers, and by powder diffraction and by solid-state NMR spectroscopy in the cases of the silver compounds. The two pairs of coordination polymers, [Cu·L-Arg·(NO_3_)_2_·H_2_O]_CP_ and [Cu·DL-Arg·(NO_3_)_2_·H_2_O]_CP_, and [Cu·L-Hys·(NO_3_)_2_·H_2_O]_CP_ and [Cu·DL-His·(NO_3_)_2_·H_2_O]_CP_, have been shown to be isostructural in spite of the different chirality of the amino acid ligands. A similar structural analogy could be established for the silver complexes on the basis of SSNMR. The activity against the bacterial pathogens *Pseudomonas aeruginosa*, *Escherichia coli*, and *Staphylococcus aureus* was assessed by carrying out disk diffusion assays on lysogeny agar media showing that, while there is no significant effect arising from the use of enantiopure or chiral amino acids, the coordination polymers exert an appreciable antimicrobial activity comparable, when not superior, to that of the metal salts alone.

## 1. Introduction

The overuse of antimicrobials during the last half-century is the primary cause of the development of antimicrobial resistance in pathogenic and opportunistic microorganisms, which has become one of the most important challenges in pharmacology and modern medicine [1,2]. Since metal-based antimicrobials have the potential for new sustainable solutions for health [3,4], progress has been made in the area of molecular inorganic–organic hybrid compounds [5,6]. Indeed, the coordination of organic ligands with metals, showing antibacterial activities, has proved to be a valid route to tackle antimicrobial resistance (AMR) [7,8,9]. Attention has been focused on the design of new bioactive metal–organic frameworks (bioMOFs) [10,11], coordination polymers [12,13], salts [14,15,16], and complexes [17,18]. These materials are engineered in order to combine a bactericidal metal center with bioactive ligands or with ancillary ligands selected from the GRAS (*generally recognized as safe*) list [19].

In this work, we extend the quest for novel antimicrobials to the investigation of a series of novel coordination polymers of copper nitrate and silver nitrate with the amino acids arginine and histidine, reacted as both enantiopure L and racemic DL forms. Hereafter, we elaborate on the reasons for choosing these metals and the amino acids as ligands.

The coinage metals copper and silver have a history of medicinal use through antiquity. It is worth recalling that silver is presently widely used in wound dressings and medical devices because it exerts broad-spectrum antimicrobial activity against Gram-positive and -negative bacteria, viruses, fungi, and protozoa [20,21], while copper is an esteemed antimicrobial agent and has long been used for its medical properties [22,23]. Nowadays, copper is widely studied in different forms and formulations for the antimicrobial treatment of surfaces [24,25] and on medical devices and implants [26,27]. We can find various examples of copper used in its metallic state [28,29,30], in a nanoparticle form [31], and as a coordination compound or ionic co-crystal [32,33,34]. The mechanism of action of silver and copper, although not fully established, consists of the disruption of cell membranes, enzymes, and nucleic acids, and interfering with cell division, inducing an oxidative stress response involving endogenous ROS (reactive oxygen species), thus leading to cell death [35,36,37,38,39,40,41].

Even though one does not regularly associate amino acids with antimicrobial properties, many antimicrobials are built upon amino acid scaffolds or incorporate amino acids into their structures [42]. Additionally, some studies have shown antimicrobial activity in particular forms [43]. Arginine is also a key building block of antimicrobial peptide structures and performs best in a polymeric configuration [44]. Histidine also acts as an antimicrobial in peptides, but requires a lower pH in order to be sure that the imidazole side chain holds a positive charge [45]. A report of efficacy against *Helicobacter pylori* suggests that its major mechanism of antimicrobial activity is the inhibition of cell wall synthesis [46].

Many examples of complexes and coordination compounds with silver and other metals (zinc, copper) [32,33,47,48] showing antimicrobial activity are available in the literature that have *basic* amino acids (arginine, histidine) as ligands [49,50,51] or compounds with N-protonated functionalities (imidazole, guanidine) [52,53,54]. In 2015, the structure of a coordination polymer with silver nitrate and the amino acid L-arginine (IWOFUX [Ag·L-Arg·NO_3_·0.5H_2_O]_CP_ [55]) was reported, showing a remarkable activity against two Gram-negative bacteria (*E. coli*, *P. aeruginosa*). Moderate activity was observed against molds (*A. niger*, *P. citrinum*) and some activity was observed for the yeasts *C. albicans*, *S. cerevisiae*, although it was not significant [51]. The structure of a silver nitrate complex with L-histidine in its zwitterionic form was also described in 2012 (TIGHEY [Ag·*bis*(L-His)·NO_3_·0.5H_2_O]_CP_ [56]) while discussing a pH-dependent and selective behavior of amino acids coordinating silver ions.

Here, we extend on our theme of combining antimicrobial metals with organic molecules in coordination polymers. In this case, we also took the opportunity to use both enantiopure and racemic forms of the amino acids with the aim of ascertaining whether the bacteriostatic activity could show differences attributable to chirality. All coordination polymers as bulk materials were investigated in terms of antimicrobial activity by the standard Kirby–Bauer disk diffusion zone of inhibition assay [57] against *Pseudomonas aeruginosa*, *Escherichia coli*, and *Staphylococcus aureus*. It can be anticipated, but vide infra, that, while no significant difference could be detected in terms of antimicrobial behavior between enantiopure and racemic products, all compounds are active against these bacterial strains. Moreover, this study has enabled us to find a remarkable structural analogy between enantiopure and racemic coordination polymers, which will be discussed hereafter.

## 2. Results and Discussion

Mechanochemical and solution methods (see below) were applied to prepare a family of compounds, which, as will be explained below, are all characterized by the assembly of the metals and of the amino acid ligands in coordination polymers. The chemical formulae and the corresponding abbreviated names are listed in Table 1.

The copper coordination polymers were all characterized by single-crystal X-ray diffraction, whereas, in the silver cases, due to the difficulty in growing suitable single crystals, the structural characterization was based on a combination of X-ray powder diffraction (XRPD) and solid-state NMR (SSNMR) spectroscopy experiments. SSNMR is known to be highly complementary to diffraction methods, providing insight into the structural and dynamic features of solid materials. Its multinuclear approach, i.e., the possibility of probing the local environment of several NMR-active nuclei, is one of the main strengths of the technique. For instance, ^13^C CPMAS spectra can give information about the purity and the degree of crystallinity of a sample, as well as the number of independent molecules in the unit cell. Moreover, the chemical shift of carboxylic moieties is quite sensitive to the protonation state of the acid group, or to the involvement of the carboxyl in the formation of hydrogen bonds, allowing for the distinction between salts and co-crystals [58]. Multinuclear SSNMR measurements have been carried out on the silver compounds listed above, as well as on the previously reported silver coordination polymers IWOFUX ([Ag·L-Arg·NO_3_·0.5H_2_O]_CP_) [55] and TIGHEY ([Ag·*bis*(L-His)·NO_3_·0.5H_2_O]_CP_) [56].

### 2.1. Structures of Enantiopure and Racemic [Cu·AA·(NO_3_)_2_]_CPs_

As pointed out in the introduction and discussed later in this manuscript, one of the purposes of this work was to establish whether the enantiopure and racemic derivatives would show a different or similar antimicrobial activity when tested against the same bacteria strains (vide infra). However, hereafter, we focus on the remarkable structural analogies between the two pairs of compounds. All of the compounds are coordination polymers in which the amino acid molecules act as bridging ligands between the metal atoms. Because of the structural similarity between the arginine and histidine compounds, it is convenient to begin with a discussion of the structures of the two pairs of copper coordination polymers, namely, the arginine compounds L-Arg·Cu and DL-Arg·Cu and the histidine compounds L-His·Cu and DL-His·Cu. These compounds were obtained by reacting copper nitrate with the L-enantiomer and the DL racemic mixture of arginine and histidine, respectively. The coordination polymers consist of amino acid moieties (in zwitterionic form) bridging consecutive copper atoms. Both histidine and arginine link metals by a bidentate-chelating Cu---N(-CHRC)O---Cu unit on one side, and by a coordination O---Cu bond on the other side. In such a way, each copper atom formally receives three electron pairs from each amino acid molecule, fulfilling a distorted octahedral coordination via two interactions with nitrate ions and with one water molecule. The close resemblance between the resulting enantiopure and racemic chains in both the arginine and histidine compounds is remarkable, even more so because the same feature is shared by both the histidine and the arginine compounds. However, in the enantiopure polymers, the bridging ligands obviously have the same chirality, while in the racemic polymers, L and D enantiomers neatly alternate in the structure, but in such a way that the polymers always show an “enantiopure side”. In other words, in the DL polymers, L and D enantiomers occupy opposite sites along the chains.

**Figure 1:**
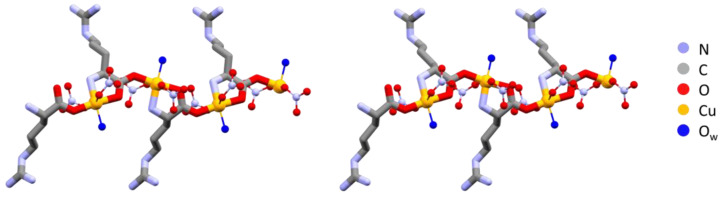
Polymeric chains in L-Arg·Cu (**left**) and DL-Arg·Cu (**right**). Note the remarkable structural similarity, with all arginine ligands having the same chirality on the left and alternate chirality on the right. Hydrogens omitted for clarity.

**Figure 2:**
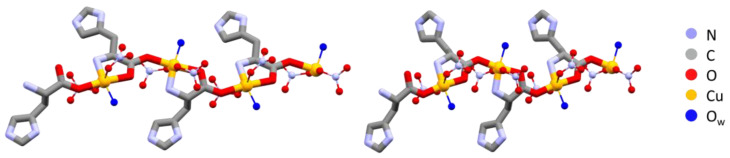
Polymeric chains in L-His·Cu form II (**left**) and DL-His·Cu (**right**). As in the case of arginine (Figure 1), the coordination polymers are structurally very similar despite the different chirality. Hydrogens omitted for clarity.

The polymeric chains in L-Arg·Cu and DL-Arg·Cu are compared in Figure 1, while those of the histidine compounds L-His·Cu and DL-His·Cu are compared in Figure 2.

**Figure 3:**
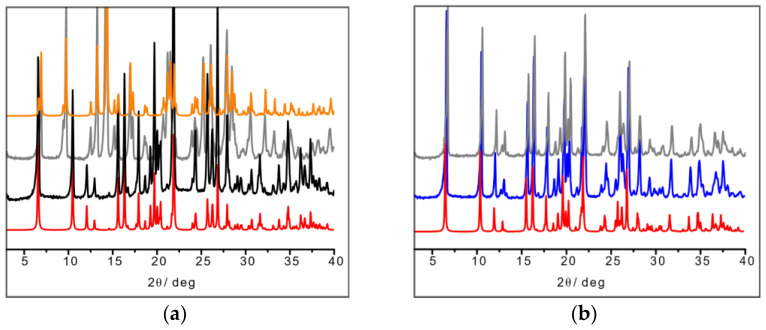
(**a**) From bottom to top: calculated pattern from single-crystal data of L-Arg·Cu (in red); powder pattern from ball milling experiment (in black); powder pattern from slurry experiment resulting in the more stable 2:1 product LAHNOX (in gray) and calculated pattern of LAHNOX [59] from database (in orange).; (**b**) From bottom to top: calculated pattern from single-crystal data of DL-Arg·Cu (in red); powder pattern from ball milling experiment (in blue) and from slurry (in gray).

In the L-arginine case, the compound obtained from ball milling and from slurry show different diffraction patterns, i.e., different compounds were obtained, which calls for an explanation. While ball milling generates the 1:1 L-arginine–copper compound for which single crystals were also obtained, the slurry procedure yielded a compound with a powder pattern corresponding to the structure of the 2:1 derivative LAHNOX [59] for which a calculated pattern could be obtained from the data available in the Cambridge Structural Database (CSD). The four patterns are compared in Figure 3a. No such difference was otherwise observed when reacting DL-arginine. Figure 3b compares the powder diffraction calculated based on the single-crystal structure of DL-Arg·Cu with those measured on the sample obtained from slurry and from ball milling.

**Figure 4:**
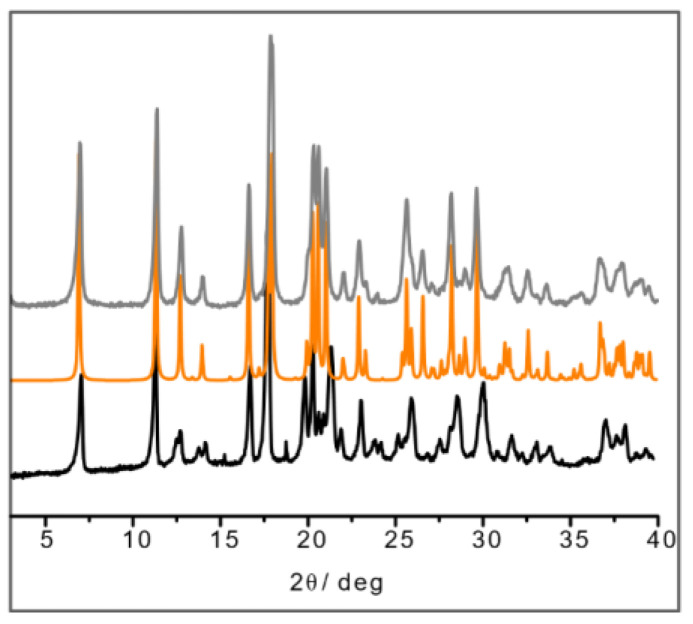
XRPD of ball-milling product of L-His·Cu (in black); calculated pattern from SCXRD of DL-His·Cu (in orange); XRPD of ball-milling product of DL-His·Cu synthesis (in gray).

The result in the case of L-His·Cu also deserves a closer look, since the crystallization product corresponding to the structure depicted in Figure 2 appears to be unstable with respect to a polymorphic transition upon manual grinding. After grinding, the powder pattern of the L-His·Cu compound closely resembles that calculated for the DL-His·Cu compound (Figure 4). It seems reasonable to conclude that the two polymorphs (I and II) of L-His·Cu are structurally very similar, with form I being the more stable phase.

### 2.2. Structures of Enantiopure and Racemic [Ag·AA·NO_3_]_CPs_

As pointed out in the Introduction, because of the paucity of structural information obtained from diffraction, we recurred to SSNMR spectroscopy to characterize the silver compounds. ^13^C, ^15^N, and ^1^H are usually the nuclei of choice to detect the presence of hydrogen bonds and evaluate their strengths, as well as to ascertain whether a protonic transfer has occurred, i.e., whether a salt has formed [60]. Specifically, in the case of ^15^N SSNMR spectra, the resonance frequency of nitrogen nuclei is significantly influenced by their partaking in protonic transfer or in the formation of hydrogen bonds. For example, the chemical shift of aliphatic nitrogen atoms increases by up to 20 ppm in the case of protonation; for an aromatic nitrogen, the change in chemical shift upon salification is much more pronounced, with a decrease of up to 120 ppm. In general, the entity of the change in the chemical shift of the signal of interest is related to the position of the proton along the hydrogen bond axis (i.e., it is less pronounced if the protonic transfer was not complete and a simple hydrogen bond was formed) [60]. Even in the case of metal coordination by a nitrogen-based moiety, the ^15^N SSNMR chemical shift proves highly informative: both aliphatic and aromatic nitrogen atoms are shielded upon donation of their lone pairs, yielding lower chemical shifts for their signals in the complex [61,62].

^13^C and ^15^N CPMAS analyses were performed in the cases of L-His·Ag, DL-His·Ag, L-His_2_·Ag [56], and DL-His_2_·Ag. The full ^13^C and ^15^N CPMAS SSNMR spectra of the coordination polymers and the pure amino acids are reported in Figure S3A–D in the Supplementary Materials; isotropic ^13^C and ^15^N SSNMR chemical shift values and J_AgN_ coupling constants for the pure amino acids and the obtained coordination polymers are reported in Table S3a,b, respectively, in the Supplementary Materials.

#### 2.2.1. L-Arg·Ag and DL-Arg·Ag

**Figure 5:**
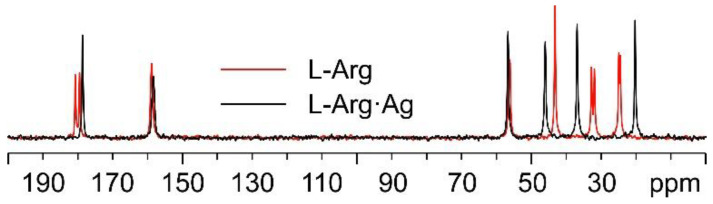
Overlay of the ^13^C CPMAS spectra of L-Arg·Ag (in black) and pure L-Arg (in red).

Starting with the ^13^C CPMAS spectra (Figure 5), the first observation is that while the spectrum of pure L-arginine (L-Arg) corresponds to that expected for the presence of two molecules in its asymmetric unit, the presence of a single set of resonances in the spectrum of L-Arg·Ag indicates that the coordination polymer only contains one amino acid molecule in the asymmetric unit. The spectrum also indicates that the crystalline powder of the obtained product corresponds to a pure compound of good crystallinity (average full width at half maximum, FWHM = 93 Hz).

**Figure 6:**
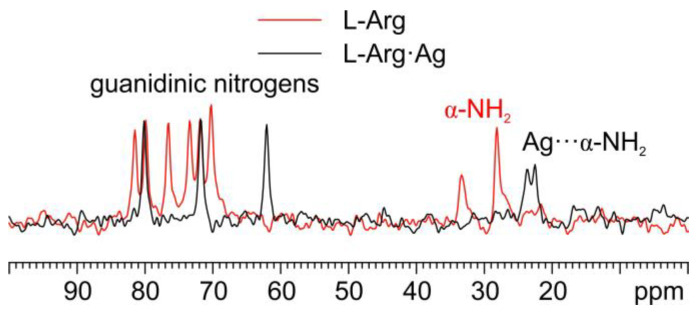
Overlay of a detail of the ^15^N CPMAS spectra of L-Arg·Ag (in black) and pure L-Arg (in red).

As for the ^15^N analyses (Figure 6), the observation of the shift towards lower frequencies of the α nitrogen signal (from 32.7 and 27.5 ppm in pure L-Arg to an average value of 23.0 ppm in L-Arg·Ag) and its splitting makes it possible to ascertain that the α-NH_2_ group of L-Arg is the one coordinating Ag.

**Figure 7:**
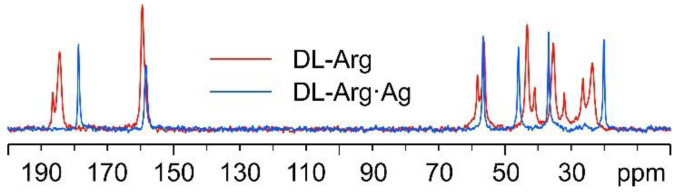
Overlay of the ^13^C CPMAS spectra of DL-Arg·Ag (in blue) and pure DL-Arg (in red).

In Figure 7, the ^13^C CPMAS spectra of DL-Arg·Ag and pure DL-arginine (DL-Arg) are displayed. The overlay demonstrates that DL-Arg·Ag is a new pure and moderately crystalline (average FWHM = 95 Hz) phase, different from the pure AA.

**Figure 8:**
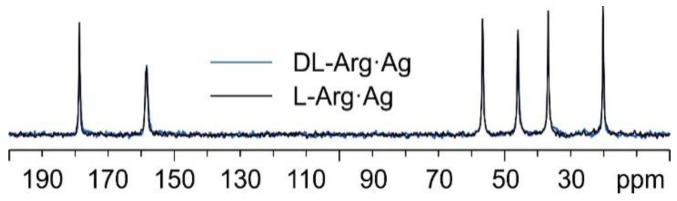
Overlay of the ^13^C CPMAS spectra of L-Arg·Ag (in black) and DL-Arg·Ag (in blue).

Interestingly, as can be observed from Figure 8, the ^13^C spectral features of DL-Arg·Ag coincide with those of L-Arg·Ag; this suggests that the two CPs are isomorphous, which may well be indicative of the formation of a conglomerate of crystals of L-Arg·Ag and of D-Arg·Ag. Indeed, this agrees with a possible spontaneous resolution of the DL-Arg·Ag racemic compound into crystals of L-Arg·Ag and of D-Arg·Ag, giving the same spectral features as those of enantiopure L-Arg·Ag. This likeness would also apply to the X-ray powder diffraction patterns.

**Figure 9:**
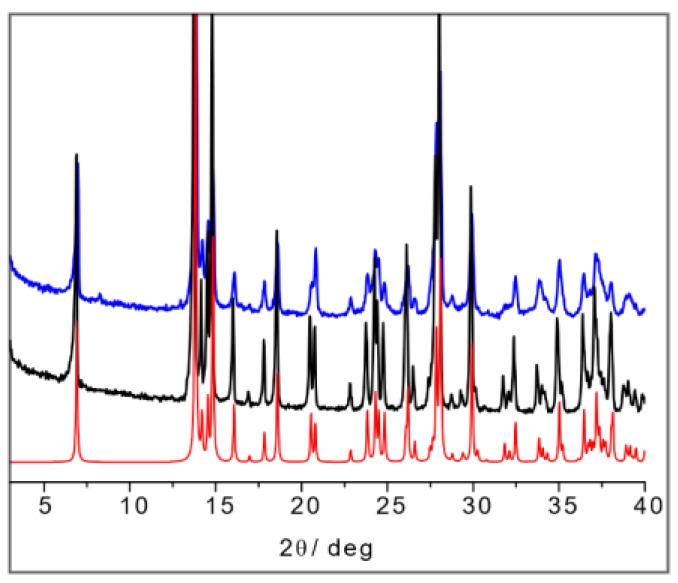
From bottom to top: calculated pattern from single-crystal data of IWOFUX (L-Arg·Ag) in red), experimental powder pattern of L-Arg·Ag (in black), and experimental powder pattern.

The comparison of the powder diffraction patterns measured on DL-Arg·Ag and on L-Arg·Ag supports this hypothesis. As can be seen in Figure 9, the patterns coincide and correspond to the pattern calculated on the basis of the single-crystal structure of L-Arg·Ag, for which data are available in the CSD.

#### 2.2.2. L-Arg·Ag and DL-Arg·Ag

**Figure 10:**
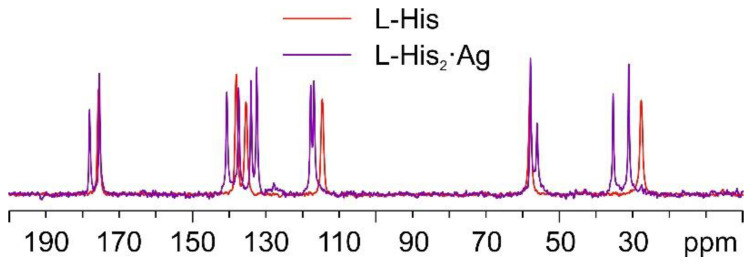
Overlay of the ^13^C CPMAS spectra of L-His_2_·Ag (in purple) and pure L-His (in red).

As mentioned above, in the case of the L-histidine complexes for the sake of the identification of the reaction products, it is necessary to consider the formation of both the 1:1 and 1:2 compounds, namely, L-His·Ag and L-His_2_·Ag. Figure 10 shows the comparison between the ^13^C CPMAS spectra of L-His_2_·Ag and pure L-histidine (L-His). A pure crystalline (average FWHM = 72 Hz) phase, different from the pure amino acid, was obtained, characterized by the presence of two independent molecules of L-His in the unit cell.

**Figure 11:**
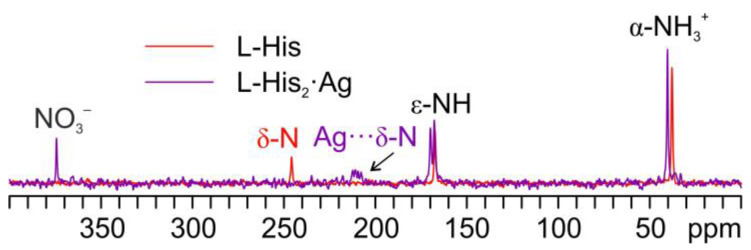
Overlay of a detail of the ^15^N CPMAS spectra of L-His_2_·Ag (in purple) and pure L-His (in red).

Figure 11 displays an overlay of the ^15^N CPMAS spectrum of L-His_2_·Ag with that of pure L-His. From the comparison, it is easy to notice how the α-NH_3_^+^ group of the pure amino acid stays charged in the coordination polymer, with the aromatic δ nitrogen being the one that binds the silver nucleus. This is proved by the shift of its ^15^N signal from 245.7 ppm in pure L-His to 211.6 and 208.5 ppm (isotropic values) in the coordination polymer, for which the signals of both molecules of L-His are split by the ^107/109^Ag-^15^N J-coupling [61].

**Figure 12:**
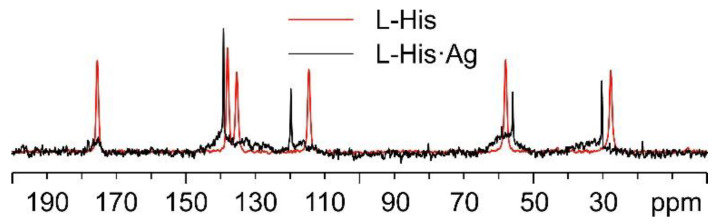
Overlay of the ^13^C CPMAS spectra of L-His·Ag (in black) and pure L-His (in red).

Regarding L-His·Ag, Figure 12 shows a comparison of its ^13^C spectrum and that of pure L-His, as for the case of L-His_2_·Ag above. As can be easily observed, the sharp signals in the spectrum of L-His·Ag fall at different chemical shifts than those of the pure amino acid. Nonetheless, very broad shoulders at the base of said peaks clearly indicate the presence of consistent amounts of amorphous material. Moreover, the weak resonances at about 20, 60, and 80 ppm can be ascribed to impurities, possibly coming from the solvents used for the synthesis. All of this confirms the sample to be quite unstable, which made the acquisition of the ^15^N CPMAS spectrum of L-His·Ag unfeasible.

**Figure 13:**
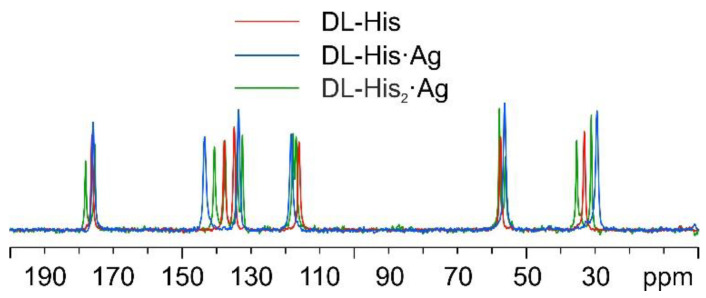
Overlay of the ^13^C CPMAS spectra of DL-His_2_·Ag (in green), DL-His·Ag (in blue), and pure DL-His (in red).

As for DL-histidine (DL-His), two pure different crystalline phases were obtained, namely, DL-His·Ag and DL-His_2_·Ag. This can be visually assessed from Figure 13, which displays their ^13^C CPMAS spectra, together with that of pure DL-His.

**Figure 14:**
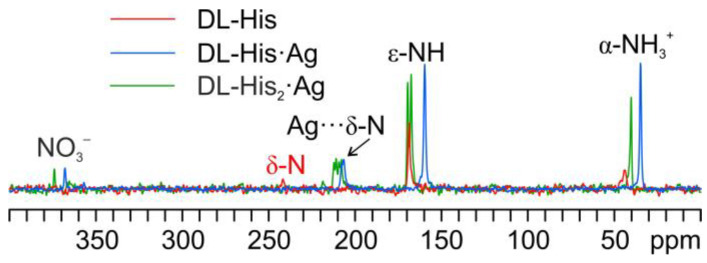
Overlay of a detail of the ^15^N CPMAS spectra of DL-His_2_·Ag (in green), DL-His·Ag (in blue), and pure DL-His (in red).

As in the other cases, the main piece of information about the identity of the coordinating N atom comes from a comparison of their ^15^N CPMAS spectra (Figure 14).

Indeed, in both coordination polymers, the δ nitrogen is the one that binds Ag, as witnessed by the chemical shifts of the corresponding signals, i.e., 207.3 ppm in DL-His·Ag and 211.6/208.5 ppm in DL-His_2_·Ag, with respect to that of the same nitrogen in pure DL-His (241.8 ppm). Moreover, in both spectra, the usual splitting of the resonances due to the coordinating N site can be observed.

**Figure 15:**
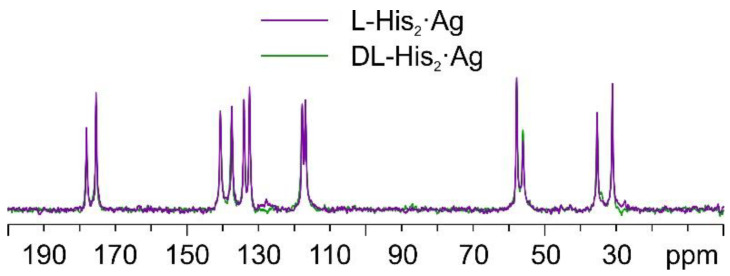
Overlay of the ^13^C CPMAS spectra of L-His_2_·Ag (in purple) and DL-His_2_·Ag (in green).

Once again, by comparing the ^13^C CPMAS spectra of L-His_2_·Ag and DL-His_2_·Ag (Figure 15), it is easy to notice that the two phases are isomorphous, as in the case of the L/DL-Arg systems.

**Figure 16:**
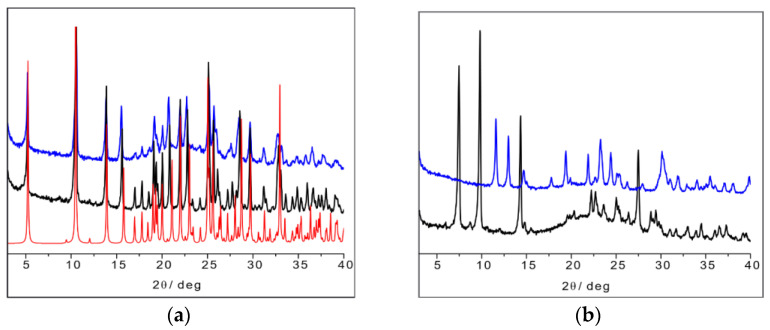
(**a**) L-His_2_·Ag (TIGHEY) [56] calculated pattern from database (in red), experimental XRPD pattern from slurry synthetic procedure of L-His_2_·Ag (in black), and DL-His_2_·Ag (in blue); (**b**) experimental XRPD pattern from slurry of L-His·Ag (in black) and DL-His·Ag (in blue).

The SSNMR information can thus be used to analyze the X-ray powder diffraction patterns in comparison with the available data from single-crystal structure determination retrieved from the CSD database. Figure 16a shows the calculated powder pattern on L-His_2_·Ag (TIGHEY) [56] in comparison with the experimental XRPD pattern measure on the product from the slurry synthetic procedure of L-His_2_·Ag (in black) and DL-His_2_·Ag (in blue). The experimental XRPD patterns of the 1:1 compounds L-His·Ag and DL-His·Ag are also compared (Figure 16b).

### 2.3. Antimicrobial Activity

The antimicrobial activity was evaluated using the established Kirby–Bauer disk diffusion assay [57]. As expected, silver nitrate gave robust antimicrobial activity with 10–15 mm zones of growth inhibition on lysogeny agar media plates. Copper nitrate provided slightly larger zones under these experimental conditions for *E. coli* and *S. aureus*. Smaller zones of inhibition were observed for each of the tested amino acids, except for L-Arg, where no inhibition was observed for the Gram-positive strain *S. aureus.* These data are shown in Figure S4A in the Supplementary Materials as zones of inhibition normalized to that of silver nitrate. Silver nitrate is a well-known antimicrobial agent and thus provides a good comparator for this study as well as an internal control to deal with plate-to-plate variation.

**Figure 17:**
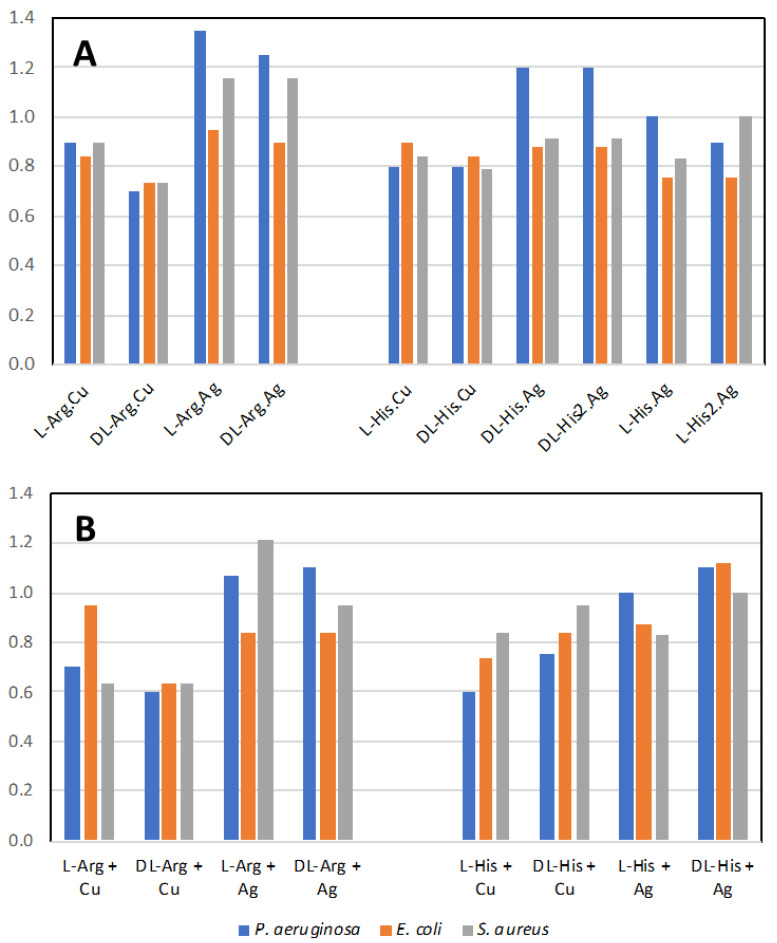
Antimicrobial activity. (**A**) Normalized antimicrobial activity from disk diffusion assay on lysogeny agar media for the coordination polymers of the metal salts with the amino acids; (**B**) mixture of the amino acid with the metal salt at 1:1 ratio. Values normalized to silver nitrate value of 1.0. Values above 1.05 are more antimicrobial than silver nitrate alone.

Figure 17A shows the normalized antimicrobial efficacy of silver nitrate and copper nitrate in combination with arginine or histidine as their coordination polymers. As a comparison, we performed direct mixing of the amino acid and the metal salt at a 1:1 ratio in order to determine whether the coordination polymer changed the antimicrobial properties (Figure 17B). Generally, there is not a large difference between the amino acids with metals presented as a combination versus a coordination polymer. Regardless, several combinations are shown to have antimicrobial activity superior to silver nitrate, particularly against *P. aeruginosa*. The variation between trials for all conditions was quite small at +/− 0.05. Thus, for these figures, one is looking for the normalized value to be >1.05 for superior antimicrobial activity and <0.95 for decreased activity, compared to silver nitrate.

In our past studies, mechanochemistry was employed to co-crystallize antimicrobial metal salts with quaternary cation compound (QCC) antiseptics, such as proflavine [15,16]. In the present study, we replaced the QCC antiseptic with the biological cation compounds of the amino acids arginine and histidine and investigated their crystal structures and properties, as well as their antimicrobial activities to three different pathogen indicator strains. The amino acids on their own have low-level antimicrobial activity compared to silver (Figure S4A). However, from the literature, we may assume that a formulation containing amino acids with side chains in their cationic state, and acting in a “polymeric” structure type, can potentially exhibit antimicrobial activity similar to cationic antimicrobial peptides (CAPs). Since our compounds have a polymeric structure, they could be considered molecular mimes of CAPs. However, overall, we observed similar activities for when the metals and amino acids were simply mixed (Figure 17B). Although we do notice a substantial enhancement of the activity for the arginine–metal coordination polymer, we should consider that a similar structure of the coordination polymer may be produced in solution, as the amino acids can generate a pseudo-molecular organic framework or metallophore complex(es).

The effect of the difference of the amino acid enantiomer is that of repressing the antimicrobial activity of the copper coordination polymer with arginine when in racemic form. The same applies to Arg^.^Ag, but only for *P. aeruginosa.* A minor effect on the antimicrobial activity was observed on comparing the racemic and enantiopure amino acid in the specific case of histidine coordination polymers with silver, particularly against *P. aeruginosa.* Combining the highly antimicrobial metal salt of copper nitrate with these amino acids reduces the antimicrobial activity. The simplest explanation for this is that the amino acid ligands form stable interactions both in solution and in the coordination polymer aggregate, thus diminishing the release of the metal ion and its bioavailability to attack the microbial cell biochemistry. On the other hand, mixing silver nitrate with the two amino acids led to superior antimicrobial activity against *P. aeruginosa* and *S. aureus*, suggesting that the amino acid ligands aid in silver ion delivery to the bacteria cells or enhance the amino acid cationic polymer character.

Further studies will tell whether the physicochemical nature of the co-crystal leads to superior ‘drug-delivery’ features over the combination of the components. There is a considerable need for biocompatible, eco-friendly, cost-effective antimicrobials with long term stability/activity in food packaging, cosmetic preservatives, and corrosion/biofouling control. It is in these areas that we envision possible applications of these and similar co-crystal compounds.

## 3. Materials and Methods

All reagents and solvents used in this work were purchased from Merck (Sigma-Aldrich) (Darmstadt, Germany) TCI Europe (Zwijndrecht, Belgium), or Fluorochem (Hadfield, UK) and then used without further purification.

### 3.1. Synthetic Procedures

All of the products were obtained using a 1:1 or 2:1 stoichiometry between the AA and the metal salts AgNO_3_ or Cu(NO_3_)_2_·3H_2_O. All of the AAs were in their anhydrous form.

#### 3.1.1. Synthesis from Slurry

[Cu·AA·(NO_3_)_2_]_CPs_ and [Ag·AA·NO_3_]_CPs_ were synthesized from slurry in ethanol (0.5 mL) and water (0.5 mL) in a 1:1 stoichiometric ratio of the reactants. For each reactant, 1 mmol was used (mass quantities reported in Table 2) and the reaction was left to stir at room temperature for 3 days in a 10 mL glass vial closed with a *PE pressure plug.* The same procedure was applied for the synthesis of the 2:1 silver-coordination compounds, in which 0.5 mmol of silver nitrate was used instead. The solid products were recovered and analyzed after filtration and drying.

#### 3.1.2. Synthesis from Ball Milling

All of the [Cu·AA·(NO_3_)_2_]_CPs_ were synthesized mechanochemically using a Retsch MM200 Mixer Mill (Verder Group, Haan, Germany), operated at a frequency of 25 Hz for 1 h, with 5 mL agate jars and 2 agate balls of 5 mm diameter, using a 1:1 mixture of ethanol and water (100 μL). The same methodology was also applied for the [Ag·AA·NO_3_]_CPs_, successfully obtaining L-Arg·Ag [55] and DL-Arg·Ag. For the [Ag·His·NO_3_]_CPs_ (L and DL), the synthesis gave slightly crystalline powders with quasi-amorphous patterns (synthesis scale 1 mmol, mass quantities as reported in Table 2). The products were left to dry out at room temperature, collected from the jar, and analyzed with XRPD.

#### 3.1.3. Synthesis from Solution

[Cu·AA·(NO_3_)_2_]_CPs_ were synthesized from a 1:1 solution of ethanol and water (1 mL) and 0.25 mmol of reactants; the mass quantities are reported in Table 3. Crystals suitable for single-crystal X-ray analysis were collected 2 weeks after the slow evaporation of the solvent mixture. All of the attempts to crystallize the [Ag·AA·NO_3_]_CPs_ series led to the reduction of silver nitrate or to the obtainment of small sized crystals that were difficult to collect and analyze.

### 3.2. Solid-State Characterization

#### 3.2.1. Single-Crystal X-ray Diffraction (SCXRD)

Structural data for [Cu·AA·(NO_3_)_2_]_CPs_ were collected at room temperature with an Oxford Diffraction X’Calibur diffractometer (Rigaku, Tokyo, Japan) equipped with a graphite monochromator and a CCD detector. The unit cell parameters for all compounds discussed herein are reported in Table S1 in the Supplementary Materials. The structures were solved by the Intrinsic Phasing methods and refined by least squares methods against F^2^ using SHELXT-2016 and SHELXL-2018 [63,64] with the Olex2 [65] interface. Non-hydrogen atoms were refined anisotropically. Hydrogen atoms were added in calculated positions. The software Mercury 2020.2.0 [66] was used to analyze and represent the crystal packing. Crystal data can be obtained free of charge via www.ccdc.cam.ac.uk/conts/retrieving.html (or from the Cambridge Crystallographic Data Centre, 12 Union Road, Cambridge CB21EZ, UK; fax: (+44)1223-336-033; or e-mail: deposit@ccdc.cam.ac.uk). CCDC 2238350-2238353.

#### 3.2.2. Powder X-ray Diffraction (XRPD)

Room-temperature powder X-ray diffraction patterns were collected on a PANalytical X’Pert Pro automated diffractometer (was PHILIPS, now Malvern PANalytical-Spectris, London, UK) equipped with an X’Celerator detector in Bragg–Brentano geometry, using Cu Kα radiation (λ = 1.5418 Å) without monochromator in the 3–40° 2θ range (step size: 0.033°; time/step: 20 s; Soller slit: 0.04 rad; anti-scatter slit: ½; divergence slit: ¼; 40 mA*40 kV).

#### 3.2.3. Variable-Temperature X-ray Powder Diffraction (VT-XRPD)

X-ray powder diffractograms in the 3–40° 2θ range were collected for L-His·Ag on a PANalytical X’Pert Pro automated diffractometer (was PHILIPS, now Malvern PANalytical-Spectris, London, UK) equipped with an X’Celerator detector and an Anton Paar TTK 450 system (Graz, Austria) for measurements at controlled temperature. Data were collected in open air in Bragg–Brentano geometry using Cu Kα radiation without a monochromator.

#### 3.2.4. Differential Scanning Calorimetry (DSC)

DSC measurements were performed for all CPs with a Perkin–Elmer Diamond instrument (Waltham, MA, USA). The samples (3–5 mg) were placed in sealed aluminum pans, and heating was carried out at 5 °C min^−1^.

#### 3.2.5. Thermogravimetric Analysis (TGA)

TGA measurements for all CPs were performed using a Perkin-Elmer TGA7 instrument (Waltham, MA, USA) in the temperature range 35–350/400 °C under an N_2_ gas flow, at a heating rate of 5 °C min^−1^. Data for DSC and TGA results are reported in the Supplementary Materials, Table S2.

#### 3.2.6. Solid-State NMR (SSNMR)

The solid-state ^13^C and ^15^N CPMAS spectra were acquired with a Jeol (Tokyo, Japan) ECZR 600 instrument, operating at 600.17, 150.91, and 60.81 MHz, respectively, for ^1^H, ^13^C, and ^15^N nuclei. The powder samples were packed into a cylindrical zirconia rotor with a 3.2 mm o.d. and a 60 μL volume. A certain amount of sample was collected from each batch and used without further preparations to fill the rotor. The ^13^C CPMAS spectra were acquired at room temperature, at a spinning speed of 20 kHz, using a ramp cross-polarization pulse sequence with a 90° ^1^H pulse of 2.0 μs and a contact time of 3.5 ms. An optimized recycle delay ranging from 1.1 to 29.7 s was used for a number of scans in the 20–22,000 range, depending on the sample. The ^15^N CPMAS spectra were acquired at room temperature, at a spinning speed of 12 kHz, using a ramp cross-polarization pulse sequence with a 90° ^1^H pulse of 2.0 μs and a contact time of 0.5, 1 or 4 ms, depending on the sample. An optimized recycle delay ranging from 1.1 to 29.7 s was used for a number of scans in the 2000–80,000 range, depending on the sample. For every spectrum, a two-pulse phase modulation (TPPM) decoupling scheme was used, with a radiofrequency field of 108.5 kHz. The ^13^C chemical shift scale was calibrated through the carboxylic signal of external standard glycine (at 176.5 ppm); the ^15^N chemical shift scale was calibrated through the signal of external standard glycine (at 33.4 ppm with reference to NH_3_).

### 3.3. Antimicrobial Activity

#### 3.3.1. Strains and Growth Medium

*Pseudomonas aeruginosa* ATCC27853, *Escherichia coli* ATCC25922, and *Staphylococcus aureus* indicator strains were used in this study. Lysogeny broth (LB) was prepared in distilled water with 10 g/L NaCl (VWR International Co., Mississauga, ON, Canada), 5 g/L yeast extract (EMD Chemicals Inc., Darmstadt, Germany), and 10 g/L tryptone (VWR Chemicals LLC, Solon, OH, USA). The agar medium for the disk diffusion assays was obtained by adding 15 g/L bacteriological agar (VWR International LLC, Solon, OH, USA) to the above-mentioned LB. Frozen cultures were revived on LB agar plates overnight at 37 °C. Colonies were transferred to liquid LB using a sterile cotton swab and incubated for 16 h at 37 °C and 150 rpm shaking, and this saturated culture was then used for the inoculant for the disk diffusion assay.

#### 3.3.2. Disk Diffusion Assay

In an aseptic environment, 150 µL of overnight culture was spread on LB agar plates per each organism and left to dry at room temperature for 1 h. Then, 25 mg/mL stock solutions or suspensions of CPs and initial compounds were prepared from powder. Controls of initial compounds were prepared as mixtures by 1:1 mixing of the 25 mg/mL stocks (i.e., 12.5 mg/mL final concentration of each compound). Kirby–Bauer blank disks were placed into a vial with 300 µL of a solution or suspension of the compound(s) of interest and left to soak for 30 min with mixing every 10 min by the inversion of the vial. The disks were then transferred to the agar culture plates (total of two replicates per compound per organism) and the plates were left in the incubator at 37 °C for 24 h, where the zone of growth inhibition was measured. Three of these biological trials were performed. To address plate-to-plate and trial variance, the average zone of inhibition measurement in mm was normalized where the zone for disk containing silver nitrate (placed on every plate) was defined as a zone of 1.0 in each biological replicate. Overall, the variance in this approach is 0.05 normalized units. This then gives an effective break point for any value >1.05 to have greater antimicrobial activity than the comparator of silver nitrate.

## 4. Conclusions

Solid formulations of metal complexes of active ingredients are being investigated for antimicrobial applications, particularly in response to the increasing threat of antimicrobial resistance. The co-crystallization of organic active molecules, such as active pharmaceutical ingredients (APIs) or naturally occurring antimicrobials or inorganic metals and complexes, has proved a fruitful way to access a great variety of new compounds, which may integrate or replace existing antimicrobials experiencing resistance. As a part of our ongoing studies, we have shown, for example, that proflavine co-crystals of metal complexes with silver, copper, zinc, and gallium can be used selectively for the best results against different bacteria strains and growth states [67].

In this paper, we investigated a family of complexes obtained by the mechanochemical and solution co-crystallization of copper and silver salts with the amino acids arginine and histidine in both enantiopure and racemic forms. The goal of this work was manifold. First, we aimed to explore the outcome of the co-crystallization of the metal salts silver nitrate and copper nitrate with arginine and histidine, which resulted in the formation of novel coordination polymers that required a combination of single-crystal and powder diffraction as well as of solid-state NMR experiments for their characterization.

In terms of structures, the two pairs of copper coordination polymers, namely, the arginine compounds L-Arg·Cu and DL-Arg·Cu and the histidine compounds L-His·Cu and DL-His·Cu, have been found to show remarkable similarities. Not only do they form isostructural coordination polymers, with the amino acid ligands bridging consecutive copper atoms, but also show that, in the DL cases, amino acids of the same chirality occupy the same side along the chains. As a consequence, the enantiopure and racemic coordination polymers are basically isostructural. Multinuclear ^15^N and ^13^C CPMAS solid-state NMR spectroscopy also revealed the same structural relationship between the L- and DL-pairs of the L/DL-Arg as well as of the L-His_2_·Ag and DL-His_2_·Ag systems.

We also evaluated the potential antibacterial activity of the resulting coordination polymers against common bacterial strains. The consequence of using both the enantiopure and the racemic amino acids in the formation of the coordination polymers was also investigated, as it was the possible presence of any “chiral preference” in the interaction of the enantiopure or racemic products with the bacteria. Even though chirality seems to add nuances to the antibacterial capacity of the compounds, our primary observation is that the amino acids arginine and histidine form coordination polymers with silver and copper, which had a level of antimicrobial activity with primary differences depending on the nature of the metal atom. Overall, this study lends further support to the idea that co-crystallization, especially in solvent-free conditions, is a cheap and effective way to explore the possibility of enhancing the properties of crystal formers, whether as a combination of active organic molecules with other organic ones or with inorganic partners. The mechanism of interactions of the coordination polymers with the bacteria membranes remains to be fully understood and will be the subject of future work.

## Figures and Tables

**Table 1:** List of the coordination polymers prepared and discussed in this work, with the respective adopted abbreviations.

[Cu·AA·(NO_3_)_2_]_CPs_	Abbreviation	[Ag·AA·NO_3_]_CPs_	Abbreviation
[Cu·L-Arg·(NO_3_)_2_·H_2_O]_CP_	L-Arg·Cu	[Ag·L-Arg·NO_3_·0.5H_2_O]_CP_ [55]	L-Arg·Ag
[Cu·DL-Arg·(NO_3_)_2_·H_2_O]_CP_	DL-Arg·Cu	[Ag·DL-Arg·NO_3_·0.5H_2_O]_CP_	DL-Arg·Ag
[Cu·L-His·(NO_3_)_2_·H_2_O]_CP_ form I	L-His·Cu form I	[Ag·L-His·NO_3_]_CP_	L-His·Ag
[Cu·L-His·(NO_3_)_2_·H_2_O]_CP_ form II	L-His·Cu form II	/	/
[Cu·DL-His·(NO_3_)_2_·H_2_O]_CP_	DL-His·Cu	[Ag·DL-His·NO_3_]_CP_	DL-His·Ag
/	/	[Ag·*bis*(L-His)·NO_3_·0.5H_2_O]_CP_ [56]	L-His_2_·Ag
/	/	[Ag·*bis*(DL-His)·NO_3_·0.5H_2_O]_CP_	DL-His_2_·Ag

**Table 2:** Mass quantities of the materials used in the solid-state synthesis (expressed in mg).

	L-Arginine	DL-Arginine	L-Histidine	DL-Histidine	AgNO_3_	Cu(NO_3_)_2_·3H_2_O
**mg**	174.20	174.20	155.16 (1:1)	155.16 (1:1)	169.87 (1:1)	241.55
**mg**	data	data	155.16 (2:1)	155.16 (2:1)	84.94 (2:1)	

**Table 3:** Mass quantities of the materials used in the solution-based synthesis (expressed in mg).

	L-Arginine	DL-Arginine	L-Histidine	DL-Histidine	AgNO_3_	Cu(NO_3_)_2_·3H_2_O
**mg**	174.20	174.20	155.16 (1:1)	155.16 (1:1)	169.87 (1:1)	241.55
**mg**	data	data	155.16 (2:1)	155.16 (2:1)	84.94 (2:1)	

## Data Availability

Data contained within this article is available in the Supplementary Materials.

## Supplementary Materials

The following supporting information can be downloaded at: https://www.mdpi.com/article/10.3390/ijms24065180/s1.

## References

[1] Antimicrobial Resistance. https://www.who.int/news-room/fact-sheets/detail/antimicrobial-resistance.

[2] Antimicrobial Resistance|European Medicines Agency. https://www.ema.europa.eu/en/human-regulatory/overview/public-health-threats/antimicrobial-resistance.

[3] Turner R.J. (2017). Metal-Based Antimicrobial Strategies. Microb. Biotechnol..

[4] Wright J.B., Lam K., Burrell R.E. (1998). Wound Management in an Era of Increasing Bacterial Antibiotic Resistance: A Role for Topical Silver Treatment. Am. J. Infect. Control.

[5] Haas K.L., Franz K.J. (2009). Application of Metal Coordination Chemistry to Explore and Manipulate Cell Biology. Chem. Rev..

[6] Lemire J.A., Kalan L., Gugala N., Bradu A., Turner R.J. (2017). Silver Oxynitrate–an Efficacious Compound for the Prevention and Eradication of Dual-Species Biofilms. Biofouling.

[7] Levy S.B., Bonnie M. (2004). Antibacterial Resistance Worldwide: Causes, Challenges and Responses. Nat. Med..

[8] Lewis K. (2011). Persister Cells: Molecular Mechanisms Related to Antibiotic Tolerance. Antibiotic Resistance. Handb. Exp. Pharmacol..

[9] Frei A., Verderosa A.D., Elliott A.G., Zuegg J., Blaskovich M.A. (2023). Metals to combat antimicrobial resistance. Nat. Rev. Chem..

[10] Pettinari C., Pettinari R., Di Nicola C., Tombesi A., Scuri S., Marchetti F. (2021). Antimicrobial MOFs. Coord. Chem. Rev..

[11] Jaros S.W., Król J., Bazanów B., Poradowski D., Chrószcz A., Nesterov D.S., Kirillov A.M., Smoleński P. (2020). Antiviral, Antibacterial, Antifungal, and Cytotoxic Silver(I) BioMOF Assembled from 1,3,5-Triaza-7-Phoshaadamantane and Pyromellitic Acid. Molecules.

[12] Jaros S.W., Guedes Da Silva M.F.C., Florek M., Smoleński P., Pombeiro A.J.L., Kirillov A.M. (2016). Silver(I) 1,3,5-Triaza-7-Phosphaadamantane Coordination Polymers Driven by Substituted Glutarate and Malonate Building Blocks: Self-Assembly Synthesis, Structural Features, and Antimicrobial Properties. Inorg. Chem..

[13] Wu F., He D., Chen L., Liu F., Huang H., Dai J., Zhang S., You J. (2018). Antibacterial Coordination Polymer Hydrogels Composed of Silver(I)-PEGylated Bisimidazolylbenzyl Alcohol. RSC Adv..

[14] Yamamoto Y., Morikawa T., Kawai T., Nonomura Y. (2017). Selective Bactericidal Activity of Divalent Metal Salts of Lauric Acid. ACS Omega.

[15] Shemchuk O., Braga D., Grepioni F., Turner R.J. (2020). Co-Crystallization of Antibacterials with Inorganic Salts: Paving the Way to Activity Enhancement. RSC Adv..

[16] Fiore C., Shemchuk O., Grepioni F., Turner R.J., Braga D. (2021). Proflavine and Zinc Chloride “Team Chemistry”: Combining Antibacterial Agents via Solid-State Interaction. CrystEngComm.

[17] Fiori A.T.M., Nakahata D.H., Cuin A., Lustri W.R., Corbi P.P. (2017). Synthesis, Crystallographic Studies, High Resolution Mass Spectrometric Analyses and Antibacterial Assays of Silver(I) Complexes with Sulfisoxazole and Sulfadimethoxine. Polyhedron.

[18] Matiadis D., Karagiaouri M., Mavroidi B., Nowak K.E., Katsipis G., Pelecanou M., Pantazaki A., Sagnou M. (2021). Synthesis and Antimicrobial Evaluation of a Pyrazoline-Pyridine Silver(I) Complex: DNA-Interaction and Anti-Biofilm Activity. BioMetals.

[19] Generally Recognized as Safe (GRAS)|FDA. https://www.fda.gov/food/food-ingredients-packaging/generally-recognized-safe-gras.

[20] Lansdown A.B.G. (2006). Silver in Health Care: Antimicrobial Effects and Safety in Use. Curr. Probl. Dermatol..

[21] Mijnendonckx K., Leys N., Mahillon J., Silver S., Van Houdt R. (2013). Antimicrobial Silver: Uses, Toxicity and Potential for Resistance. BioMetals.

[22] Mitra D., Kang E.T., Neoh K.G. (2020). Antimicrobial Copper-Based Materials and Coatings: Potential Multifaceted Biomedical Applications. ACS Appl. Mater. Interfaces.

[23] Dollwet H., Sorenson J. (2001). Historic Uses of Copper Compounds in Medicine. Trace Elements in Medicine.

[24] Casey A.L., Adams D., Karpanen T.J., Lambert P.A., Cookson B.D., Nightingale P., Miruszenko L., Shillam R., Christian P., Elliott T.S.J. (2010). Role of Copper in Reducing Hospital Environment Contamination. J. Hosp. Infect..

[25] Airey P., Verran J. (2007). Potential Use of Copper as a Hygienic Surface; Problems Associated with Cumulative Soiling and Cleaning. J. Hosp. Infect..

[26] Wang Y., Zhang W., Yao Q. (2021). Copper-Based Biomaterials for Bone and Cartilage Tissue Engineering. J. Orthop. Transl..

[27] Shen Q., Qi Y., Kong Y., Bao H., Wang Y., Dong A., Wu H., Xu Y. (2022). Advances in Copper-Based Biomaterials With Antibacterial and Osteogenic Properties for Bone Tissue Engineering. Front. Bioeng. Biotechnol..

[28] Quaranta D., Krans T., Santo C.E., Elowsky C.G., Domaille D.W., Chang C.J., Grass G. (2011). Mechanisms of Contact-Mediated Killing of Yeast Cells on Dry Metallic Copper Surfaces. Appl. Environ. Microbiol..

[29] Santo C.E., Lam E.W., Elowsky C.G., Quaranta D., Domaille D.W., Chang C.J., Grass G. (2011). Bacterial Killing by Dry Metallic Copper Surfaces. Appl. Environ. Microbiol..

[30] Grass G., Rensing C., Solioz M. (2011). Metallic Copper as an Antimicrobial Surface. Appl. Environ. Microbiol..

[31] Sánchez-Sanhueza G., Fuentes-Rodríguez D., Bello-Toledo H. (2016). Copper Nanoparticles as Potential Antimicrobial Agent in Disinfecting Root Canals: A Systematic Review. Int. J. Odontostomatol..

[32] Gungor O., Kocer F., Kose M. (2020). Cu(II) Complexes of Biguanidine Ligands: Structural Characterisation, DNA Binding and Antimicrobial Properties. J. Mol. Struct..

[33] Zalevskaya O.A., Gur’eva Y.A. (2021). Recent Studies on the Antimicrobial Activity of Copper Complexes. Russ. J. Coord. Chem. Khimiya.

[34] Grepioni F., Casali L., Fiore C., Mazzei L., Sun R., Shemchuk O., Braga D. (2022). Steps towards a nature inspired inorganic crystal engineering. Dalton Trans..

[35] Celik S., Yurdakul S., Erdem B. (2022). New Silver(I) Complex as Antibiotic Candidate: Synthesis, Spectral Characterization, DFT, QTAIM and Antibacterial Investigations and Docking Properties. J. Mol. Struct..

[36] Lansdown A.B. (2013). Silver I: Its Antibacterial Properties and Mechanism of Action. J. Wound Care.

[37] Dakal T.C., Kumar A., Majumdar R.S., Yadav V. (2016). Mechanistic Basis of Antimicrobial Actions of Silver Nanoparticles. Front. Microbiol..

[38] Hong R., Kang T.Y., Michels C.A., Gadura N. (2012). Membrane Lipid Peroxidation in Copper Alloy-Mediated Contact Killing of Escherichia Coli. Appl. Environ. Microbiol..

[39] Tian W.X., Yu S., Ibrahim M., Almonaofy A.W., He L., Hui Q., Bo Z., Li B., Xie G. (2012). lin Copper as an Antimicrobial Agent against Opportunistic Pathogenic and Multidrug Resistant Enterobacter Bacteria. J. Microbiol..

[40] Mathews S., Hans M., Mücklich F., Solioz M. (2013). Contact Killing of Bacteria on Copper Is Suppressed If Bacterial-Metal Contact Is Prevented and Is Induced on Iron by Copper Ions. Appl. Environ. Microbiol..

[41] Gutierrez H., Portman T., Pershin V., Ringuette M. (2013). Evaluation of Biocidal Efficacy of Copper Alloy Coatings in Comparison with Solid Metal Surfaces: Generation of Organic Copper Phosphate Nanoflowers. J. Appl. Microbiol..

[42] Nowak M.G., Skwarecki A.S., Milewska M.J. (2021). Amino Acid Based Antimicrobial Agents—Synthesis and Properties. ChemMedChem.

[43] Cutrona K.J., Kaufman B.A., Figueroa D.M., Elmore D.E. (2015). Role of Arginine and Lysine in the Antimicrobial Mechanism of Histone-Derived Antimicrobial Peptides. FEBS Lett..

[44] Sepahi M., Jalal R., Mashreghi M. (2017). Antibacterial Activity of Poly-l-Arginine under Different Conditions. Iran. J. Microbiol..

[45] Kacprzyk L., Rydengård V., Mörgelin M., Davoudi M., Pasupuleti M., Malmsten M., Schmidtchen A. (2007). Antimicrobial Activity of Histidine-Rich Peptides Is Dependent on Acidic Conditions. Biochim. Biophys. Acta—Biomembr..

[46] Minami M., Ando T., Hashikawa S.N., Torii K., Hasegawa T., Israel D.A., Ina K., Kusugami K., Goto H., Ohta M. (2004). Effect of Glycine on Helicobacter Pylori in Vitro. Antimicrob. Agents Chemother..

[47] Kalarani R., Sankarganesh M., Kumar G.G.V., Kalanithi M. (2020). Synthesis, Spectral, DFT Calculation, Sensor, Antimicrobial and DNA Binding Studies of Co(II), Cu(II) and Zn(II) Metal Complexes with 2-Amino Benzimidazole Schiff Base. J. Mol. Struct..

[48] Repon R., Islam T., Sadia H.T., Mikučionienė D., Hossain S., Kibria G., Kaseem M. (2021). Development of Antimicrobial Cotton-Fabric-Impregnating AgNPs Utilizing Contemporary Practice. Coatings.

[49] Kasuga N.C., Takagi Y., Tsuruta S.I., Kuwana W., Yoshikawa R., Nomiya K. (2011). Synthesis, Structure and Antimicrobial Activities of Meso Silver(I) Histidinate [Ag2(D-His)(L-His)]n (Hhis = Histidine) Showing Different Self-Assembly from Those of Chiral Silver(I) Histidinates. Inorg. Chim. Acta.

[50] Nomiya K., Takahashi S., Noguchi R., Nemoto S., Takayama T., Oda M. (2000). Synthesis and Characterization of Water-Soluble Silver(I) Complexes with L-Histidine (H2his) and (S)-(-)-2-Pyrrolidone-5-Carboxylic Acid (H2pyrrld) Showing a Wide Spectrum of Effective Antibacterial and Antifungal Activities. Crystal Structures of Chiral Helical Polymers [Ag(Hhis)](n) and {[Ag(Hpyrrld)]2}(n) in the Solid State. Inorg. Chem..

[51] Legler A.V., Kazachenko A.S., Kazbanov V.I., Per’yanova O.V., Veselova O.F. (2001). Synthesis and Antimicrobial Activity of Silver Complexes with Arginine and Glutamic Acid. Pharm. Chem. J..

[52] Abuskhuna S., Briody J., McCann M., Devereux M., Kavanagh K., Fontecha J.B., McKee V. (2004). Synthesis, Structure and Anti-Fungal Activity of Dimeric Ag(I) Complexes Containing Bis-Imidazole Ligands. Polyhedron.

[53] Nomiya K., Tsuda K., Sudoh T., Oda M. (1997). Ag(I)-N Bond-Containing Compound Showing Wide Spectra in Effective Antimicrobial Activities: Polymeric Silver(I) Imidazolate. J. Inorg. Biochem..

[54] Kasuga N.C., Yoshikawa R., Sakai Y., Nomiya K. (2012). Syntheses, Structures, and Antimicrobial Activities of Remarkably Light-Stable and Water-Soluble Silver Complexes with Amino Acid Derivatives, Silver(I) N-Acetylmethioninates. Inorg. Chem..

[55] Ahmad S., Yousaf A., Tahir M.N., Isab A.A., Monim-Ul-Mehboob M., Linert W., Saleem M. (2016). Structural Characterization and Antimicrobial Activity of a Silver(I) Complex of Arginine. J. Struct. Chem..

[56] Mirolo L., Schmidt T., Eckhardt S., Meuwly M., Fromm K.M. (2013). PH-Dependent Coordination of AgI Ions by Histidine: Experiment, Theory, and a Model for SilE. Chem. Eur. J..

[57] Balouiri M., Sadiki M., Ibnsouda S.K. (2016). Methods for in Vitro Evaluating Antimicrobial Activity: A Review. J. Pharm. Anal..

[58] Aramini A., Bianchini G., Lillini S., Bordignon S., Tomassetti M., Novelli R., Mattioli S., Lvova L., Paolesse R., Chierotti M.R. (2021). Unexpected Salt/Cocrystal Polymorphism of the Ketoprofen–Lysine System: Discovery of a New Ketoprofen–l-Lysine Salt Polymorph with Different Physicochemical and Pharmacokinetic Properties. Pharmaceuticals.

[59] Hu R., Yu Q., Liang F., Ma L., Chen X., Zhang M., Liang H., Yu K. (2008). Syntheses and Crystal Structures of Cis—And Trans-Copper(II) Complexes of L-Arginine. J. Coord. Chem..

[60] Cerreia Vioglio P., Chierotti M.R., Gobetto R. (2017). Pharmaceutical Aspects of Salt and Cocrystal Forms of APIs and Characterization Challenges. Adv. Drug Deliv. Rev..

[61] Daolio A., Pizzi A., Calabrese M., Terraneo G., Bordignon S., Frontera A., Resnati G. (2021). Molecular Electrostatic Potential and Noncovalent Interactions in Derivatives of Group 8 Elements. Angew. Chemie Int. Ed..

[62] Pettinari R., Marchetti F., Pettinari C., Condello F., Skelton B.W., White A.H., Chierotti M.R., Gobetto R. (2016). Self-Assembly of Arene Ruthenium Acylpyrazolone Fragments to Tetranuclear Metallacycles. Molecular Structures and Solid-State 15N CPMAS NMR Correlations. Dalt. Trans..

[63] Sheldrick G. (2014). SHELXT: Integrating Space Group Determination and Structure Solution. Acta Crystallogr. Sect. A Found. Adv..

[64] Sheldrick G.M. (2015). SHELXT—Integrated Space-Group and Crystal-Structure Determination. Acta Crystallogr. Sect. A Found. Crystallogr..

[65] Dolomanov O.V., Bourhis L.J., Gildea R.J., Howard J.A.K., Puschmann H. (2009). OLEX2: A Complete Structure Solution, Refinement and Analysis Program. J. Appl. Crystallogr..

[66] MacRae C.F., Sovago I., Cottrell S.J., Galek P.T.A., McCabe P., Pidcock E., Platings M., Shields G.P., Stevens J.S., Towler M. (2020). Mercury 4.0: From Visualization to Analysis, Design and Prediction. J. Appl. Crystallogr..

[67] Lekhan A., Fiore C., Shemchuk O., Grepioni F., Braga D., Turner R.J. (2022). Comparison of Antimicrobial and Antibiofilm Activity of Proflavine Co-crystallized with Silver, Copper, Zinc, and Gallium Salts. ACS Appl. Biomat..

