# Supplementary material for: Mechanochemical Preparation, Solid-State Characterization, and Antimicrobial Performance of Copper and Silver Nitrate Coordination Polymers with L- and DL-Arginine and Histidine
Source: Int J Mol Sci. 2023 Mar 8;24(6):5180. doi: 10.3390/ijms24065180 (PMC10049651; doi:10.3390/ijms24065180)
Supplement: Supplementary file 1 [file ijms-24-05180-s001.zip › ijms-2213594-supplementary.pdf]

Supplementary material

Mechanochemical preparation, solid-state characterization and antimicrobial performance of copper and silver nitrate coordination polymers with L-and DL-arginine and histidine.

CONTENTS

- S1. CRYSTALLOGRAPHIC DATA
- S2. TGA AND DSC DATA
- S3. SOLID-STATE NMR SUPPORTING DATA
- S4. ANTIMICROBIAL ACTIVITY SUPPORTING DATA

Supplementary material

S1. Crystallographic Data

Table S1. Crystallographic table for data obtained from single-crystal XRD.

	L-Arg·Cu	DL-Arg·Cu	L-His·Cu	DL-His·Cu
**Chemical formula**	$C_6H_{14}N_6O_8Cu·H_2O$	$C_6H_{14}N_6O_8Cu·H_2O$	$C_6H_9N_5O_8Cu·H_2O$	$C_6H_9N_5O_8Cu·H_2O$
**M_r /g mol^{-1}**	379.79	379.79	720.61	360.74
**Crystal system**	Monoclinic	Monoclinic	Monoclinic	Monoclinic
**Space group**	$P2_1$	$P2_1/c$	$P2_1$	$P2_1/c$
**a / Å**	10.0142 (2)	13.4092 (7)	9.9964 (4)	12.9972 (12)
**b / Å**	10.7768 (2)	10.7584 (4)	9.9530 (4)	9.8254 (5)
**c / Å**	13.4634 (3)	10.0233 (4)	12.2937 (5)	10.0294 (8)
**α / °**	90	90	90	90
**β / °**	100.950 (2)	100.181 (4)	97.009 (4)	104.187 (8)
**γ / °**	90	90	90	90
**V / Å^3**	1426.53 (5)	1423.21 (11)	1214.02 (8)	1241.72 (17)
**Z, Z'**	4, 2	4, 1	2, 1	4, 1
**d / mg cm^{-3}**	1.768	1.773	1.970	1.930
**μ / mm^{-1}**	1.59	1.59	1.86	1.82
**Measd reflns**	11944	6492	6121	9894
**Indep reflns**	6517	3294	4125	3018
**Reflns with $I > 2σ(I)$**	5706	2581	3613	2732
**R_{int}**	0.026	0.031	0.035	0.033
**$R1$ [$F^2 > 2σ(F^2)$]**	0.042	0.050	0.062	0.100
**$wR(F^2)$**	0.075	0.121	0.163	0.240

Supplementary material

S2. TGA and DSC data

Table S2. TGA and DSC results.

L-Arg·Ag (IWOFUX)

TGA

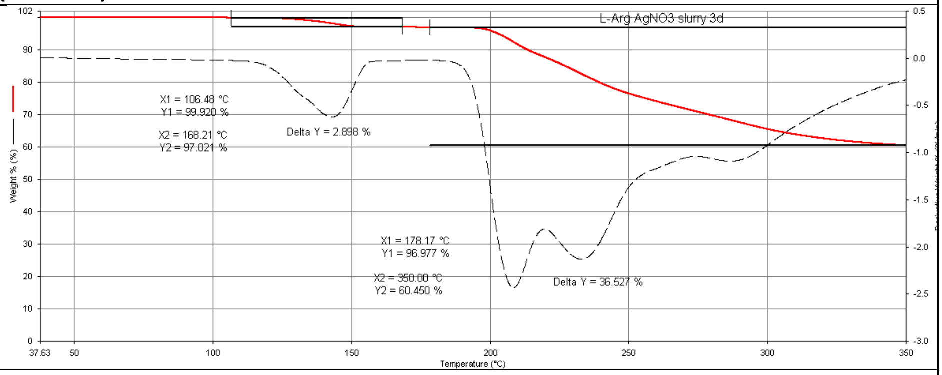

DSC

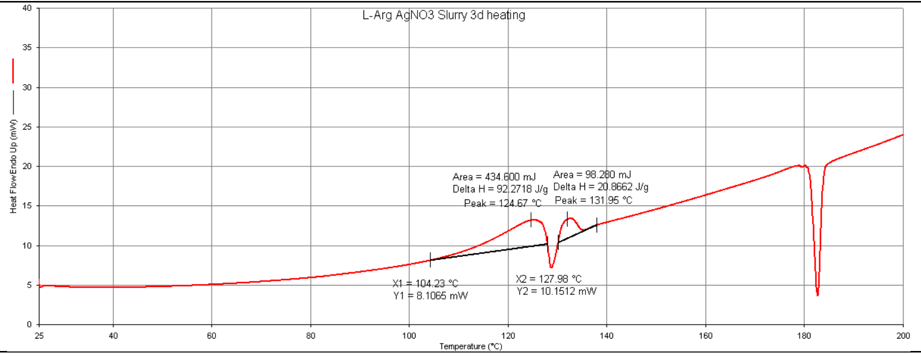

DL-Arg·Ag

TGA

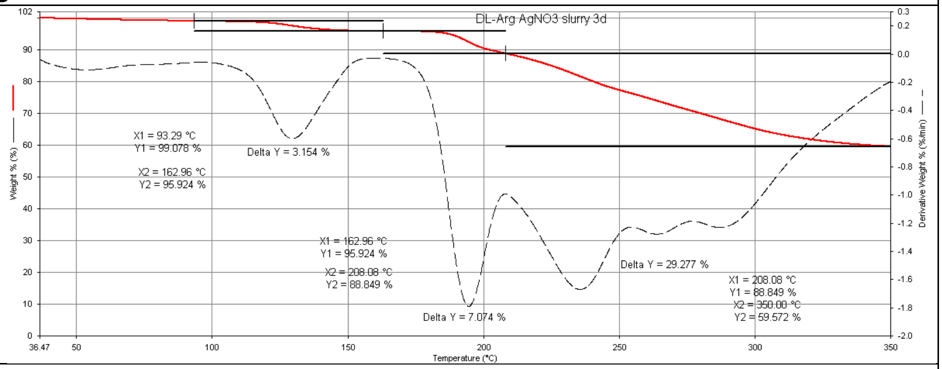

DSC

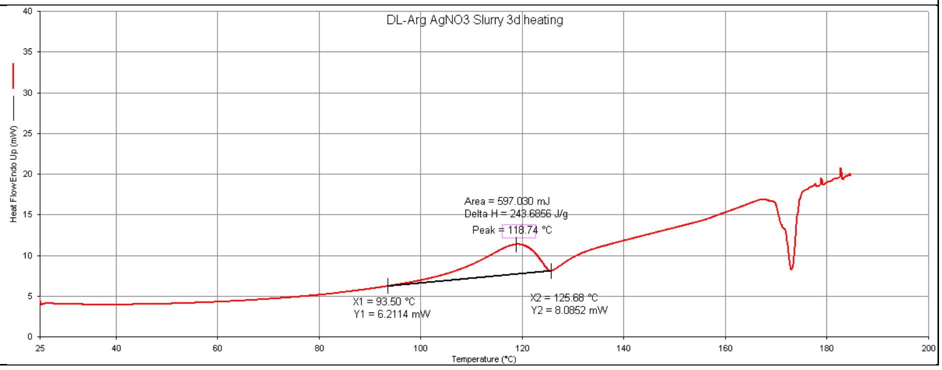

Supplementary material

L-His$_2$·Ag (TIGHEY)

TGA

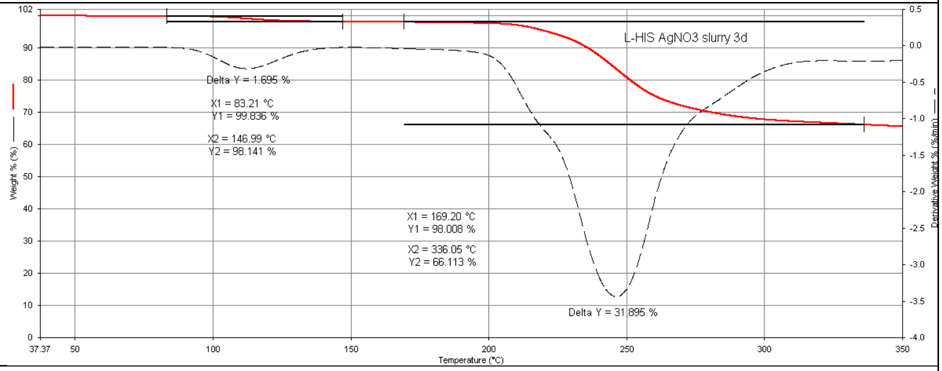

DSC

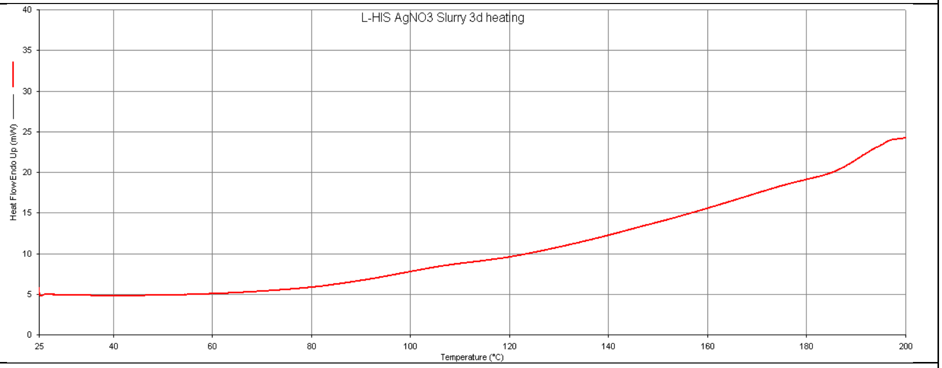

DL-His$_2$·Ag

TGA

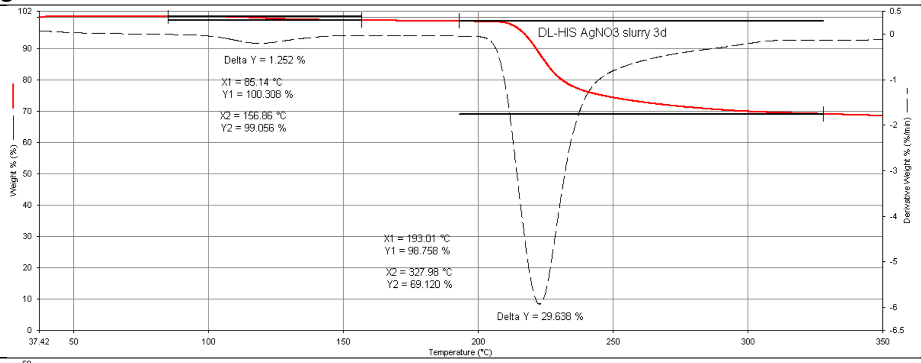

DSC

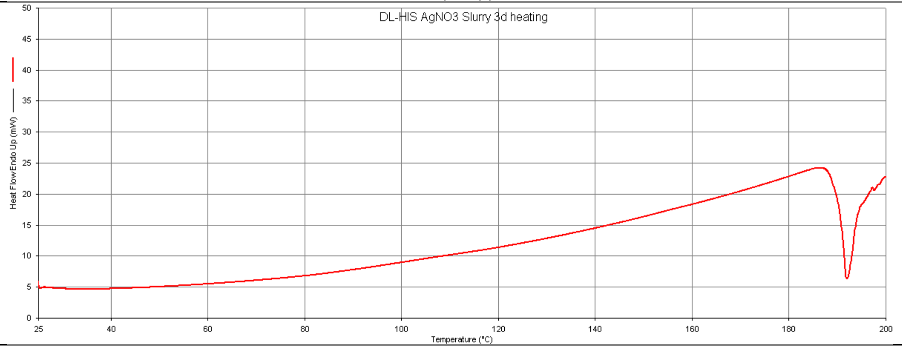

L-His·Ag

Supplementary material

TGA

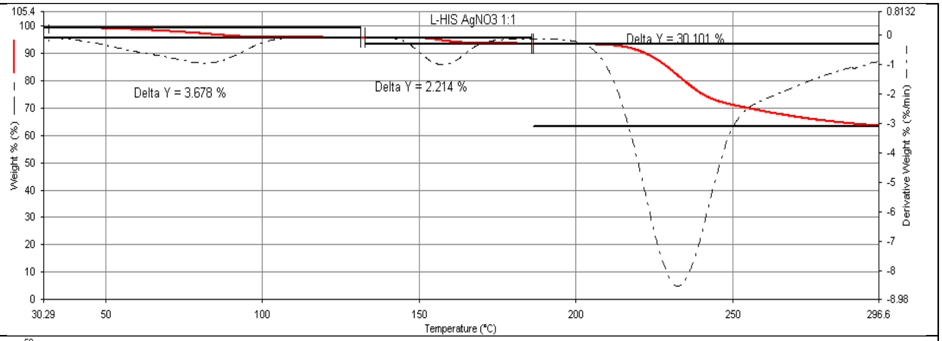

DSC

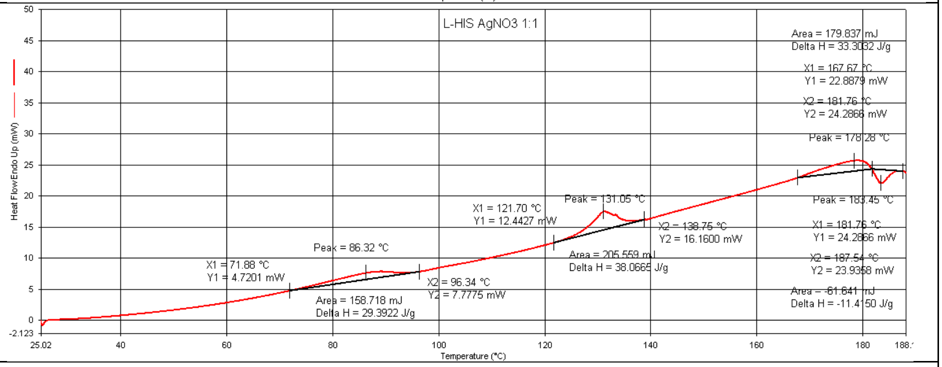

DL-His·Ag

TGA

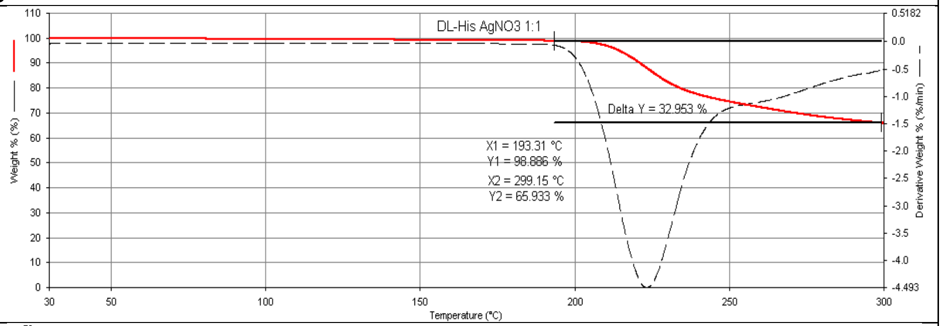

DSC

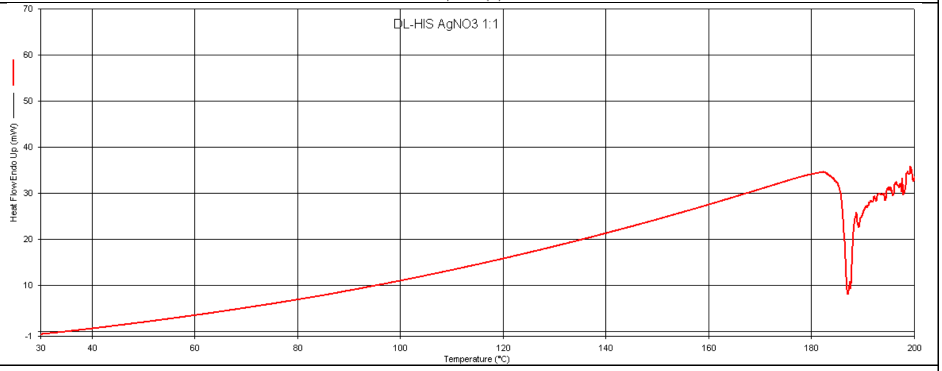

L-Arg·Cu

TGA

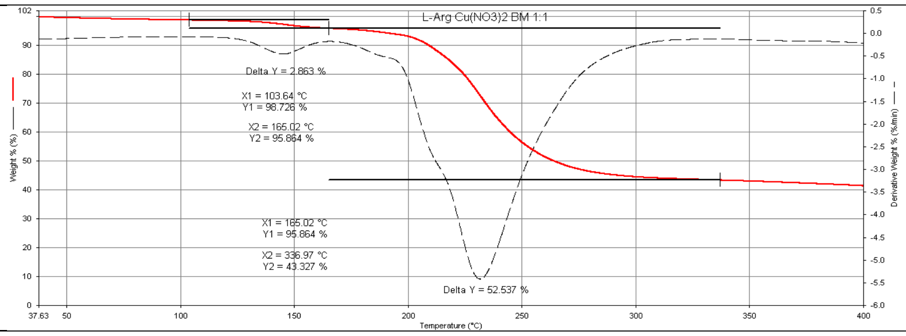

Supplementary material

DSC

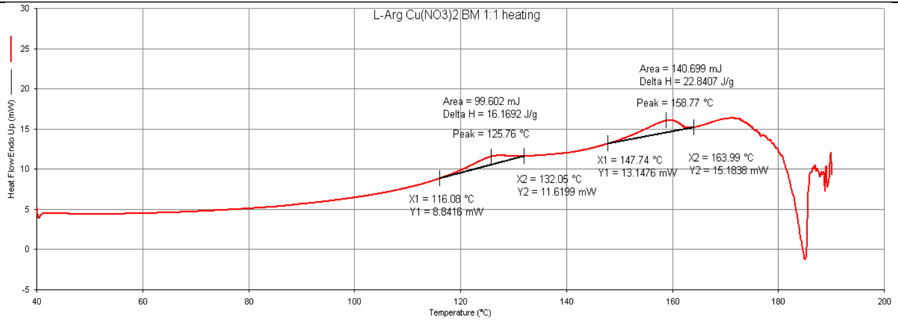

DL-Arg·Cu

TGA

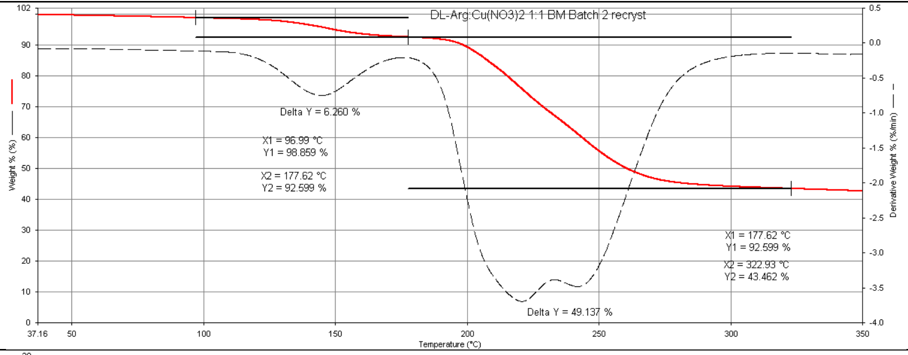

DSC

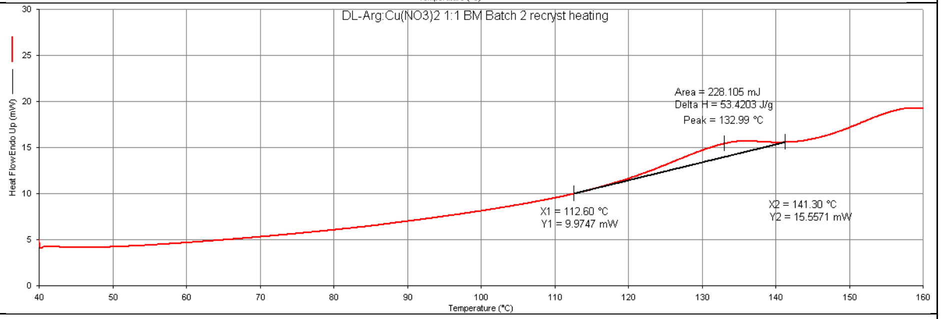

L-His·Cu

TGA

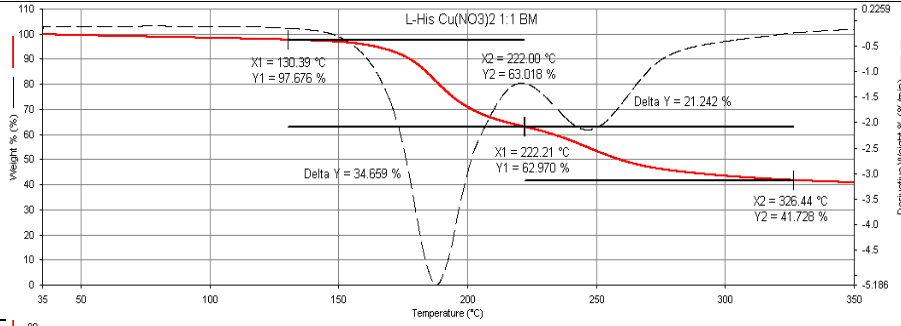

DSC

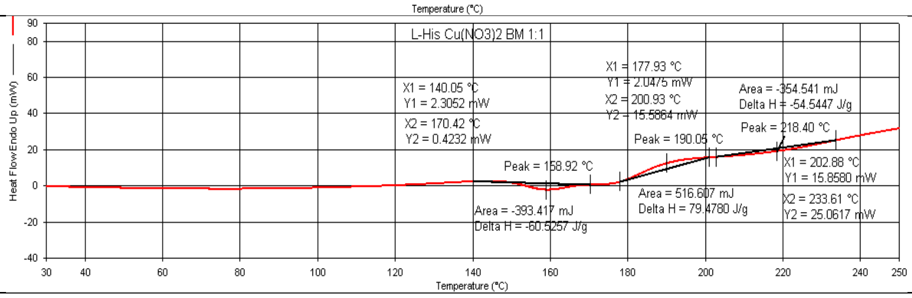

DL-His·Cu

Supplementary material

<table>
<tr><td>TGA</td><td>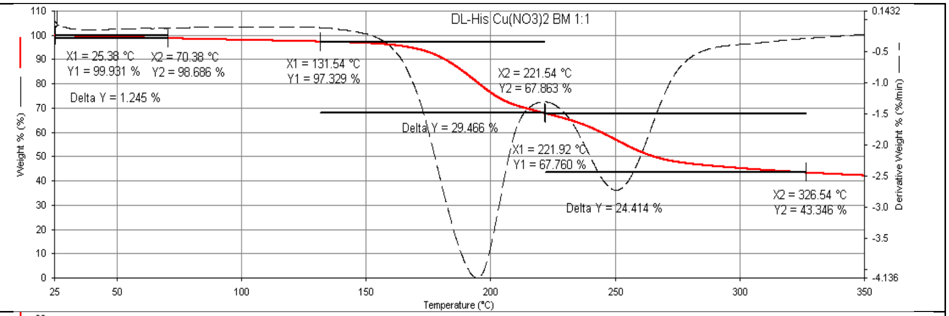
</td></tr>
<tr><td>DSC</td><td>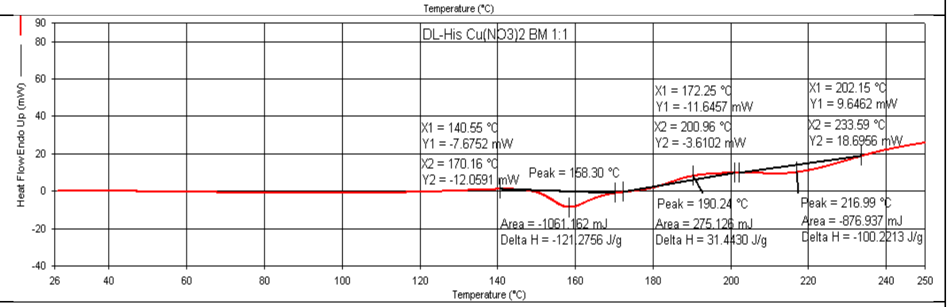
</td></tr>
<tr><td></td><td></td></tr>
</table>

S3. Solid-state NMR

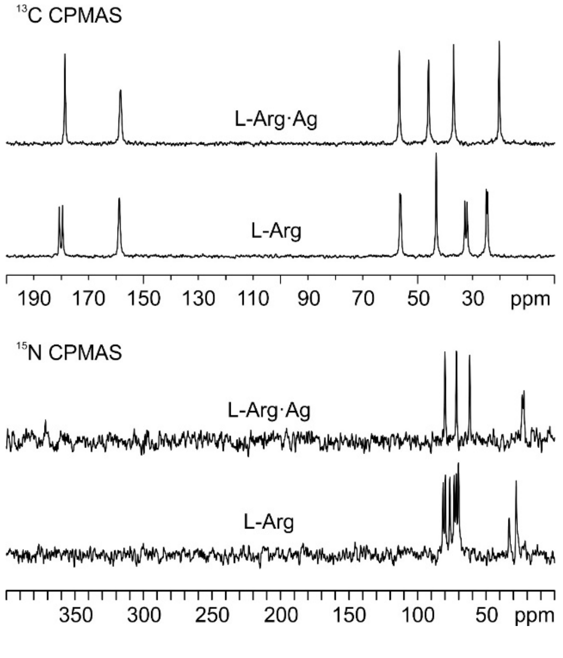

Figure S3A. Top: ^{13}C (150.9 MHz) CPMAS spectra of L-Arg·Ag and pure L-Arg, acquired at a spinning speed of 20 kHz at room temperature; bottom: ^{15}N (60.8 MHz) CPMAS spectra of L-Arg·Ag and pure L-Arg, acquired at a spinning speed of 12 kHz at room temperature.

Supplementary material

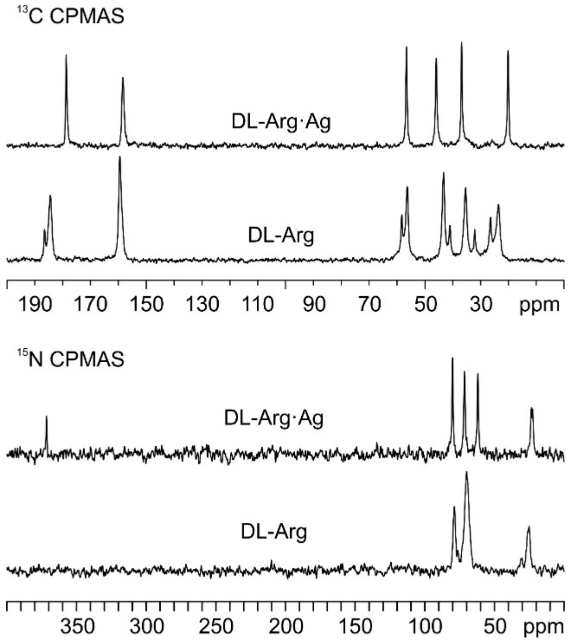

Figure S3B. Top: ^{13}C (150.9 MHz) CPMAS spectra of DL-Arg·Ag and pure DL-Arg, acquired at a spinning speed of 20 kHz at room temperature; bottom: ^{15}N (60.8 MHz) CPMAS spectra of DL-Arg·Ag and pure DL-Arg, acquired at a spinning speed of 12 kHz at room temperature.

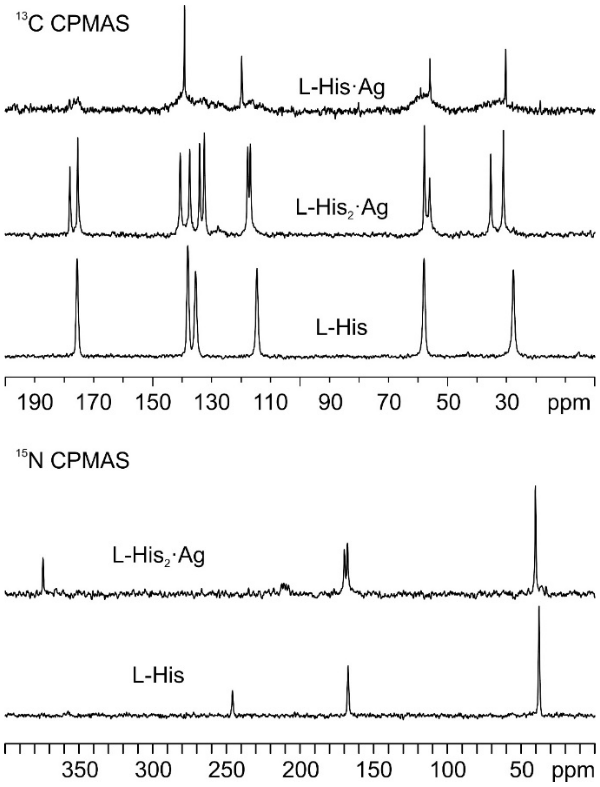

Figure S3C. Top: ^{13}C (150.9 MHz) CPMAS spectra of L-His·Ag, L-His_2·Ag and pure L-His, acquired at a spinning speed of 20 kHz at room temperature; bottom: ^{15}N (60.8 MHz) CPMAS spectra of L-His_2·Ag and pure L-His, acquired at a spinning speed of 12 kHz at room temperature.

Supplementary material

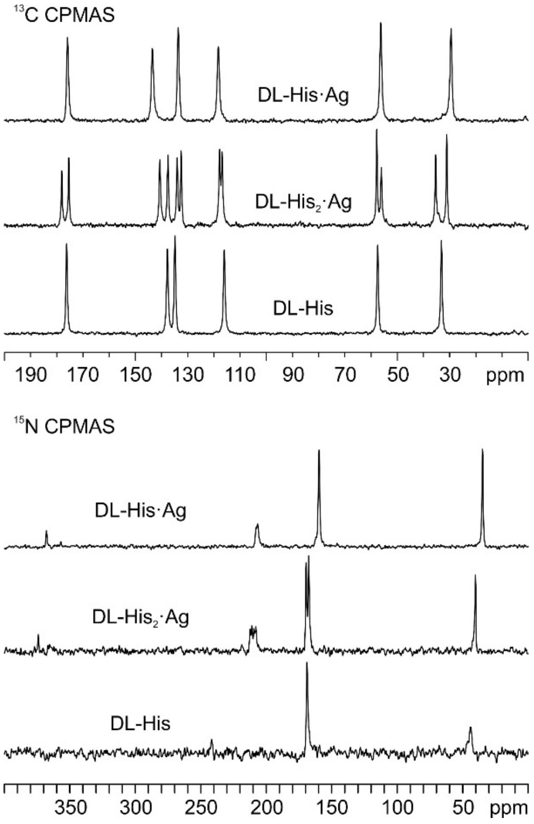

Figure S3D. Top: ^{13}C (150.9 MHz) CPMAS spectra of DL-His·Ag, DL-His$_2$·Ag and pure DL-His, acquired at a spinning speed of 20 kHz at room temperature; bottom: ^{15}N (60.8 MHz) CPMAS spectra of DL-His·Ag, DL-His$_2$·Ag and pure DL-His, acquired at a spinning speed of 12 kHz at room temperature.

Table S3a. ^{13}C and ^{15}N SSNMR isotropic chemical shift values (in ppm) of the peaks observed in the CPMAS spectra of the employed AAs.

L-Arg	DL-Arg	L-His	DL-His
**^{13}C SSNMR chemical shift (ppm)**			
179.9	186.5	175.6	176.2
178.7	184.3	138.1	137.7
158.1	159.5	135.4	134.9
55.6	58.3	114.6	116.0
55.3	56.3	58.0	57.5
42.3	43.3	27.7	33.1
31.9	41.0		
31.1	35.4		
24.0	32.1		
23.6	26.4		
	23.6		
**^{15}N SSNMR chemical shift (ppm)**			
80.6	78.9	245.7	241.8
79.0	70.1	167.3	168.9
75.8	25.2	37.8	43.8
72.6			
71.1			
69.5			
32.7			
27.5			

Supplementary material

Table S3b. ^{13}C and ^{15}N SSNMR isotropic chemical shift values (in ppm) of the peaks observed in the CPMAS spectra of the achieved coordination polymers. When observed, J_{AgN} coupling constants (Hz) are reported in parentheses as averaged values between J_{109AgN} and J_{107AgN}.

L-Arg·Ag	DL-Arg·Ag	L-His·Ag	L-His$_2$·Ag	DL-His·Ag	DL-His$_2$·Ag
^{13}C SSNMR chemical shift (ppm)					
178.7	178.7	178.3	178.1	175.9	178.1
158.3	158.3	139.2	175.4	143.5	175.4
56.7	56.6	119.8	140.6	133.6	140.6
45.9	45.9	55.9	137.4	118.3	137.5
36.8	36.8	30.3	134.1	56.3	134.0
20.2	20.2		132.5	29.4	132.5
			117.8		117.8
			116.9		116.9
			57.8		57.8
			56.1		56.0
			35.3		35.3
			31.1		31.1
^{15}N SSNMR chemical shift (ppm)					
371.6	371.6	/	374.3	368.0	374.1
80.1	80.1	/	211.6 (91)	207.3 (73)	211.6 (83)
71.7	71.5	/	208.5 (96)	159.7	208.7 (83)
62.0	62.0	/	169.9	34.8	169.6
23.0 (70)	23.1 (59)		167.7		167.6
			40.2		40.2

S4. Antimicrobial activity

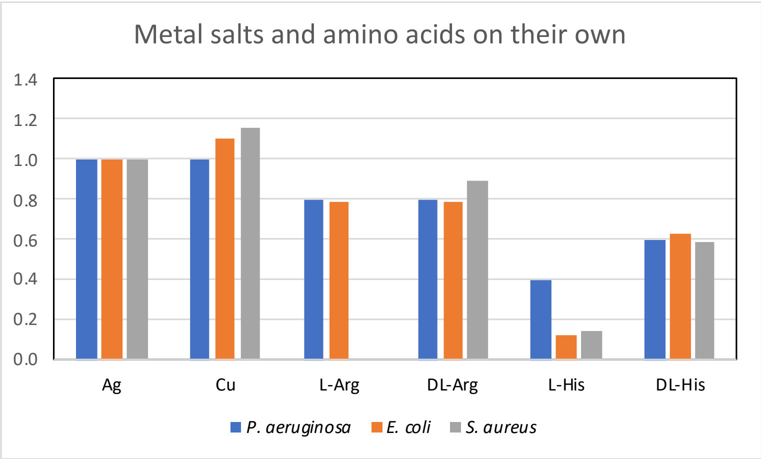

Figure S4A. Normalized antimicrobial activity of compounds used in this study on their own as obtained by disk diffusion experiments on lysogeny media agar media. Values normalized to silver nitrate value of 1.0. Values above 1.05 are more antimicrobial than silver. N = 3.
